# Depletion of acidic phospholipids influences chromosomal replication in *Escherichia coli*

**DOI:** 10.1002/mbo3.46

**Published:** 2012-11-16

**Authors:** Nicholas Fingland, Ingvild Flåtten, Christopher D Downey, Solveig Fossum-Raunehaug, Kirsten Skarstad, Elliott Crooke

**Affiliations:** 1 Department of Biochemistry and Molecular & Cellular Biology, Georgetown University Medical CenterWashington DC, 20057; 2 Department of Cell Biology, Institute for Cancer Research, Norwegian Radium Hospital, Oslo University Hospital0310, Oslo, Norway; 3 Department of Chemistry and Biochemistry, University of ColoradoBoulder, Colorado, 80309; 4 Lombardi Comprehensive Cancer Center, Georgetown University Medical CenterWashington DC, 20057

**Keywords:** Anionic phospholipids, chromosomal replication, DnaA, *E. coli*, *oriC*

## Abstract

In *Escherichia coli*, coordinated activation and deactivation of DnaA allows for proper timing of the initiation of chromosomal synthesis at the origin of replication (*oriC*) and assures initiation occurs once per cell cycle. *In vitro*, acidic phospholipids reactivate DnaA, and *in vivo* depletion of acidic phospholipids, results in growth arrest. Growth can be restored by the expression of a mutant form of DnaA, DnaA(L366K), or by *oriC*-independent DNA synthesis, suggesting acidic phospholipids are required for DnaA- and *oriC-*dependent replication. We observe here that when acidic phospholipids were depleted, replication was inhibited with a concomitant reduction of chromosomal content and cell mass prior to growth arrest. This global shutdown of biosynthetic activity was independent of the stringent response. Restoration of acidic phospholipid synthesis resulted in a resumption of DNA replication prior to restored growth, indicating a possible cell-cycle-specific growth arrest had occurred with the earlier loss of acidic phospholipids. Flow cytometry, thymidine uptake, and quantitative polymerase chain reaction data suggest that a deficiency in acidic phospholipids prolonged the time required to replicate the chromosome. We also observed that regardless of the cellular content of acidic phospholipids, expression of mutant DnaA(L366K) altered the DNA content-to-cell mass ratio.

## Introduction

Initiation of bacterial chromosomal replication is a highly regulated process that ensures replication occurs only once per cell cycle. In *Escherichia coli*, DnaA plays a central role in initiation, forming a multimeric oligomer at the origin of replication (*oriC*), unwinding origin duplex DNA, and helping direct the assembly of a replisome. DnaA exists in two forms: the replicatively active ATP-bound form and the replicatively inactive ADP-bound form (Sekimizu et al. 1987). The distribution between these two forms varies as cells progress through the cell cycle (Kurokawa et al. 1999).

Initiation of replication is triggered by ATP-DnaA binding to key determinant sites within *oriC*, which leads to proper multimerization of DnaA and subsequent melting of *oriC* (Speck et al. 1999; McGarry et al. 2004; Leonard and Grimwade 2011). To prevent reinitiation within the same cell cycle, several negative regulatory processes shut down the capacity of DnaA to initiate DNA replication: (i) conversion of DnaA-bound ATP to ADP through a process known as Regulatory Inactivation of DnaA, or RIDA (Kato and Katayama 2001; Camara et al. 2003; Kurz et al. 2004; Camara et al. 2005; Kawakami et al. 2006; Riber et al. 2006), (ii) sequestration of newly replicated *oriC* DNA by SeqA protein (Lu et al. 1994; von Freiesleben et al. 1994; Slater et al. 1995; Riber and Løbner-Olesen, 2005; Nievera et al. 2006; Waldminghaus and Skarstad 2009), and (iii) titration of DnaA from *oriC* by binding to the *datA* locus (Kitagawa et al. 1996, 1998; Katayama et al. 2001; Morigen et al. 2001; Ogawa et al. 2002). These processes are balanced by events that increase the cellular concentration of active DnaA, including expression of newly synthesized DnaA (Kurokawa et al. 1999), and reactivation of ADP-DnaA through its association with the DnaA Reactivation Sequences (DARS), DARS1 and DARS2 (Fujimitsu and Katayama 2004; Fujimitsu et al. 2009).

Moreover, interaction of ADP-DnaA with acidic phospholipids can reactivate ADP-DnaA *in vitro* (Sekimizu and Kornberg 1988; Crooke et al. 1992; Castuma et al. 1993). The exchange of ADP for ATP bound to purified DnaA is slow, with a half-life of approximately 30 min (Sekimizu and Kornberg 1988). However, when ADP-DnaA is exposed to acidic phospholipids in a fluid bilayer, release of bound nucleotide is rapid (Sekimizu and Kornberg 1988), and if ADP-DnaA is associated with *oriC* and physiological levels of ATP are present, treatment with an acidic fluid membrane causes exchange of DnaA-bound ADP for ATP, thus rejuvenating DnaA (Sekimizu and Kornberg 1988; Crooke et al. 1992; Castuma et al. 1993; Crooke 2001; Boeneman and Crooke 2005).

The *E. coli* inner membrane is primarily composed of zwitterionic phosphatidylethanolamine (∼70%) and the anionic phospholipids phosphatidylglycerol (∼25%) and cardiolipin (∼4%) (Raetz 1986). Both acidic phospholipid species, cardiolipin and phosphatidylglycerol, are synthesized through a common biosynthetic pathway that involves phosphatidylglycerol phosphate synthase A (*pgsA*). In the *E. coli* strain, MDL12 expression of the chromosomal copy of *pgsA* relies on the inducer β-d-1-thiogalactopyranoside (IPTG) (Xia and Dowhan 1995). In the absence of the inducer, the concentration of acidic phospholipids decrease as cells undergo successive rounds of division, until a threshold level is reached and growth is arrested. The arrested cells remain viable and can resume growth following addition of IPTG (Xia and Dowhan 1995).

The growth arrest caused by deficient levels of acidic phospholipids can be suppressed by the deletion of *rnhA* (Xia and Dowhan 1995) via *recA*-dependent constitutive stable DNA replication (cSDR), which bypasses normal *oriC*-dependent initiation (Kogoma and von Meyenburg 1983). This restoration of growth by cSDR suggests a close link between normal membrane lipid composition and *oriC*-based initiations of chromosomal replication.

Suppression of growth arrest also can be achieved by expression of DnaA harboring a single-point mutation, DnaA(L366K) (Zheng et al. 2001), within a region of DnaA previously identified as important for DnaA–acidic membrane interaction (Garner and Crooke 1996; Garner et al. 1998). Interestingly, overexpression of wild-type DnaA cannot restore growth under acidic phospholipid-depleted conditions (Zheng et al. 2001), suggesting that DnaA(L366K) does not compensate for a simple lack of DnaA activity in acidic phospholipid-depleted cells. The mechanism of how DnaA(L366K) suppresses the growth arrest remains unclear, although it is known that DnaA(L366K) can be reactivated by acidic phospholipids *in vitro* (Li et al. 2005), only occupy high-affinity binding sites at *oriC* whether ADP or ATP bound (Saxena et al. 2011), and is a feeble initiator of replication, and thus unable to serve as the only form of DnaA in the cell (Zheng et al. 2001; Li et al. 2005). By whatever mechanism, the ability of DnaA(L366K) to restore growth to acidic phospholipid-deficient cells suggests an intriguing relationship between acidic phospholipids and DnaA-dependent initiation of chromosomal replication.

We demonstrate here through flow cytometry that depletion of cellular acidic phospholipids was accompanied by inhibited initiation. The deficiency in acidic phospholipids resulted in a concomitant shutdown of DNA replication and protein synthesis, with this global shutdown unrelated to the stringent response. Upon restoration of acidic phospholipid synthesis, growth-arrested cells underwent a period of increased DNA replication followed by a step-wise increase in cell number, indicating a possible cell-cycle-specific arrest had occurred when the cellular concentration of acidic phospholipids dropped below a threshold level. Moreover, in addition to affecting initiation events, the depletion of acidic phospholipids appeared to prolong the time required to complete replication of the chromosome. Expression of mutant DnaA(L366K), in addition to restoring growth to acidic phospholipid-deficient cells as previously seen, decreased the DNA content-to-cell mass ratio, in agreement with other findings that DnaA(L366K) is a feeble initiator (Zheng et al. 2001; Li et al. 2005; Saxena et al. 2011).

## Experimental Procedures

### Media, strains, and plasmids

Bacterial cells were grown at 30°C with shaking in LB, M9 (Miller 1972), or morpholinopropane sulfonate (MOPS) (Neidhardt et al. 1974) media supplemented as indicated. Strain MG1655 was used as a wild-type *E. coli* K12 strain. Strain CF1651 is MG1655(*relA251::kan*) (Metzger et al. 1989; Gaal and Gourse 1990); strain MDL12 is MG1655 (*pgsA30::kan* φ[*lacOP-pgsA*^*+*^]*1 lacZ' lacY*::Tn9) (Xia and Dowhan 1995). Plasmid pZL607 contains the gene for DnaA(L366K) under control of the arabinose promoter (Zheng et al. 2001). All media for cells transformed with pZL607 contained ampicillin (100 μg/mL). Expression of DnaA (L366K) was repressed by the inclusion of glucose (0.2%) in the media and induced by addition of arabinose (0.2%). Serine hydroxamate, rifampicin, and cephalexin were from Sigma Aldrich (St. Louis, MO). IPTG and polyethyleneimine (PEI) cellulose thin-layer chromatography plates were from Thermo Fisher Scientific, Inc. (Waltham, MA). [^32^P]-orthophosphate (9100 Ci/mmol) and [^3^H-methyl]-thymidine (83.2 Ci/mmol) were from Perkin Elmer, Inc. (Waltham, MA).

To confirm the IPTG dependence of MDL12 cells for growth, glycerol stocks were streaked onto LB agar plates that contained kanamycin (50 μg/mL) and that also did or did not contain IPTG (1 mmol/L). Colonies from candidate streaks exhibiting IPTG-dependent growth were used to inoculate liquid media (rich or minimal, as indicated below) that contained kanamycin (50 μg/mL) and that also did or did not contain IPTG (10 μmol/L), and the cultures were grown overnight. Only cells that were dependent on IPTG for growth overnight were used further (the concentration of IPTG in the overnight cultures was at 10 μmol/L so that a consistent relatively rapid onset of arrested growth occurred when the cells subsequently were shifted to media lacking IPTG). To repress the synthesis of acidic phospholipids, cells were harvested from overnight cultures, washed three times with IPTG-free medium to remove residual IPTG, and then used to inoculate fresh medium containing kanamycin (50 μg/mL).

### Flow cytometric analysis of chromosomal replication

IPTG-dependent MDL12 cells were used to inoculate LB medium (25 mL) that contained kanamycin (50 μg/mL) and either did or did not contain IPTG (1 mmol/L). MDL12/pZL607 cells were cultured in medium that also contained ampicillin (100 μg/mL) and either with glucose (0.2%) or with arabinose (0.2%). Cell growth was assessed by measurement of optical density (OD_600nm_). To maintain exponential growth, when optical densities approached 0.2, cultures were diluted to an optical density of 0.01 in fresh, prewarmed LB medium (25 mL) that contained the original respective supplements. To prepare ethanol-fixed cells (termed “exponential”) for flow cytometry, cells were harvested from aliquots (5 mL) of cultures, resuspended in TE buffer, pH 8.0 (1 mL) again collected by centrifugation, resuspended in TE buffer, pH 8.0 (100 μL), and immediately mixed with 77% sterile, ice-cold ethanol (1 mL). A second sample (5 mL) of culture was harvested in parallel and mixed (3 h, 30°C) with cephalexin (100 μg/mL) and rifampicin (300 μg/mL). At the end of the 3-h treatment with cephalexin and rifampicin, the cells were fixed with ethanol as described above for the “exponential” cells. All samples are then stored at 4°C in the dark until analyzed as described previously (Torheim et al. 2000).

### Analysis of *in vivo* (p)ppGpp synthesis

Cells were grown at 30°C in minimal MOPS minimal medium (Neidhardt et al. 1974) that contained glucose (0.4%), thiamine (1 μg/mL), and the 20 amino acids (each at 20 μg/mL); for cultures of cells treated with serine hydroxamate, serine was omitted from the medium. Additionally, for CF1651 and MDL12 cells, the medium contained kanamycin (50 μg/mL). Acidic phospholipid synthesis was induced in MDL12 cells, where indicated, by inclusion of IPTG (1 mmol/L). Overnight cultures were used to inoculated fresh medium (2 mL) to an optical density (OD_600nm_) of 0.025 and [^32^P]-orthophosphate (100 μCi/mL) was added. To assess the ability of cells to induce the stringent response, a culture of each strain, MG1655, CF1651, and MDL12, was grown at 30°C for approximately three generations, then samples (0.2 mL) were collected before and after treatment for 10 min with serine hydroxamate (500 μg/mL). To assess whether depletion of acidic phospholipids induced the stringent response, cultures of MDL12 cells were grown with or without IPTG (1 mmol/L) and samples (0.2 mL) were collected at indicated times. Samples were vigorously mixed with ice-cold formic acid (20 μL; 11 mol/L) and maintained on ice. An aliquot (20 μL) of a 5:4:1 mixture (400 mmol/L NaWO_4_, 500 mmol/L TEA-Cl, 500 mmol/L procaine-HCl) was added to each sample, followed by vigorous mixing. Insoluble material was removed by centrifugation (16,000*g*, 15 min, 4°C). A portion (equivalent to 5 μL of culture at OD_600nm_ of 0.6) of the supernatant for each sample was spotted on a PEI cellulose thin-layer chromatography plate, which was then soaked for 1 min in methanol (100%) to remove water and salts. Nucleotides were separated using potassium phosphate monobasic (1.5 mol/L; pH 3.5) as the mobile phase, and visualized with a Storm 840 Phosphorimager (Molecular Dynamics, Sunnyvale, CA). Densitometric analysis was performed using NIH ImageJ software (version 1.39). A Student's *t*-test was conducted to determine statistical significance between samples.

### Measurement of radiolabeled thymidine incorporation

MDL12 cells confirmed to be IPTG-dependent for growth were used to inoculate to an optical density (OD_600nm_) of 0.01 M9 minimal medium (100 mL) containing glucose (0.1%), casamino acids (0.2%), kanamycin (50 μg/mL), and with or without IPTG (1 mmol/L). Cell growth was monitored by measurements of optical density (OD_600nm_), and at 6 h cells from both cultures were harvested by centrifugation and resuspended in fresh, prewarmed medium (50 mL) to an optical density (OD_600nm_) of 0.025. A portion of the cells that were grown for the 6 h in the absence of IPTG were resuspended in medium that contained IPTG (1 mmol/L).

Periodically, portions (200 μL) of each culture were removed, mixed with Isoflow Sheath Fluid (Beckman Coulter, Brea, CA) (800 μL), and three aliquots were counted using a Hausser 3200 hemocytometer (Hausser Scientific, Horsham, PA), with the triplicate values averaged to calculate the concentration of cells (cells/mL). In parallel, a portion (1 mL) of each culture was mixed with [^3^H-methyl]-thymidine (2 μCi/mL; 83.2 Ci/mmol) and incubated for 3 min (30°C). Radiolabeling was stopped by the addition of ice-cold trichloroacetic acid to a final concentration of 10% and the sample was retained on ice. Acid-insoluble material was collected by vacuum filtration onto GF/C filters (Millipore, MA) that had been previously soaked in a solution of 1 mol/L HCl and 100 mmol/L sodium pyrophosphate. The filters were washed twice with a solution of 1 mol/L HCl and 100 mmol/L sodium pyrophosphate, twice with 100% ethanol, dried under a heating lamp, and radioactivity measured by liquid scintillation counting. Following the last time point of the experiment, dependence on IPTG for growth was confirmed for each culture by streaking a sample onto appropriate solid growth media.

### quantitative polymerase chain reaction analysis of *oriC* and *ter* loci dosage

MDL12 cells confirmed to be IPTG-dependent for growth were used to inoculate to an optical density (OD_600nm_) of 0.01 M9 minimal medium (100 mL) containing glucose (0.1%), casamino acids (0.2%), kanamycin (50 μg/mL), and with or without IPTG (1 mmol/L). Cell growth was monitored by measurement of optical density (OD_600nm_), and cultures periodically diluted with fresh, prewarmed media to maintain exponential growth. At 6 h, the culture lacking IPTG was split and inducer added (1 mmol/L IPTG) to one portion. Periodically, aliquots (4 mL) were collected from each culture for qPCR analysis. Cells were collected by centrifugation, resuspended in TE buffer, pH 8.0 (1 mL), and again pelleted. The supernatant was removed, the cells resuspended in TE buffer, pH 8.0 (100 μL), and 77% ice-cold ethanol (1 mL) was added.

To determine the *oriC*-to-*ter* ratios by qPCR, chromosomal DNA was purified from fixed samples. Fixed cells were collected by centrifugation and lysed with 1.2% sodium dodecyl sulfate and 4 mmol/L EDTA (65°C for 5 min). DNA was precipitated with 0.7 volumes of isopropanol and washed with 70% ethanol. The DNA was treated with RNaseA (8.3 units/mL) and proteinase K (1 unit/mL) (Sigma-Aldrich, St. Louis, MO) for 45 min (37°C) and 1 h (37°C), respectively, and then proteins were precipitated with Protein Precipitation Solution (200 μL) (Promega, Fitchburg, WI). The DNA was subsequently precipitated with isopropanol and collected by centrifugation. Samples of DNA (5–10 ng) were mixed with 1× TaqMan Gene Expression mix (20 μL) (Applied Biosystems, Foster City, CA). The primers used for the qPCR amplification were 5′GAGAATATGGCGTACCAGCA and 5′-AAGACGCAGGTATTTCGCTT-3′ for amplification of the *oriC* region and 5′-TCCTCGCTGTTTGTCATCTT-3′ and 5′-GGTCTTGCTCGAATCCCTT-3′ for amplification of the *ter* region. The fluorescent probes were 5′ Fam - 3′ Tamra with the sequence 5′-CAACCTGACTTCGGTCCGCG and 5′-CATCAGCACCCACGCAGCAA-3′ for *oriC* and *ter*, respectively. The data from the samples were normalized to the data obtained from MG1655 wild-type cells treated with rifampicin and cephalexin for 2 h so that they have an *oriC*-to-*ter* ratio of 1:1.

## Results

### The loss of acidic phospholipids results in inhibited initiation of chromosomal replication and cells with decreased DNA content and cell mass

Previous studies suggested a link between acidic phospholipids and *oriC-* and DnaA-dependent chromosomal replication. To determine the *in vivo* influence of acidic phospholipids on chromosomal replication, flow cytometry was utilized to compare the chromosomal content and cell mass of cells synthesizing and not synthesizing acidic phospholipids. Controlled acidic phospholipid production was possible using strain MDL12 (Xia and Dowhan 1995).

**Figure 1:**
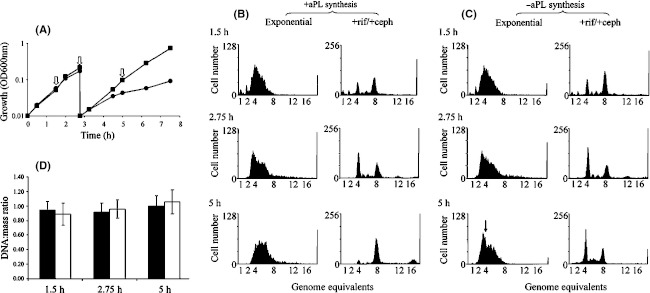
(A) MDL12 cells were grown at 30°C in LB medium that contained kanamycin (50 μg/mL) and without (•) or with (▪) IPTG (1 mmol/L). Open arrows indicate times that samples were collected for flow cytometry. At 2.75 h, the cultures were diluted with prewarmed fresh media to maintain exponential growth. (B and C) Flow cytometry histograms from cells synthesizing acidic phospholipids (B) or not synthesizing acidic phospholipids (C). Left columns are for exponential samples; right columns are for rifampicin- and cephalexin-treated samples. In panel C, the black arrow in the exponential histogram of the 5-h sample highlights the accumulation of cells with four fully replicated chromosomes. (D) DNA content-to-cell mass ratios, determined from exponential samples, are an average of the ratio at a given time point, derived from three independent experiments. Error bars represent one standard deviation. Black and white bars are for cells induced and uninduced for *pgsA* expression, respectively.

Cells (MDL12) cultured overnight in LB medium in the presence of IPTG (10 μmol/L) were used to inoculate media with and without IPTG (1 mmol/L), and the subsequent cell growth was monitored (Fig. 1A). A similar method to prepare cultures was used in subsequent experiments unless otherwise stated. Initially, the two cultures had similar growth rates, and the average doubling time under these conditions was 45 min (Table S1). As growth continued, cultures were diluted at 2.75 h with fresh, prewarmed media to decrease the cultures to an optical density (OD_600nm_) of 0.01 to ensure the cells could maintain exponential growth. Cells synthesizing acidic phospholipids continued to grow at the same rate. However, after approximately 4 h, the growth rate of cells not synthesizing acidic phospholipids gradually began to arrest (Fig. 1A).

Samples for flow cytometry analysis were collected at 1.5, 2.75, and 5 h from both cultures (Fig. 1A, open arrows). The samples were either treated for two to three generations with rifampicin and cephalexin and then fixed with ethanol or untreated and fixed immediately with ethanol, and termed “exponential” samples. Rifampicin- and cephalexin-treated cells are able to complete ongoing rounds of replication, but cannot initiate new rounds nor divide. Thus, in the flow cytometry profiles of rifampicin- and cephalexin-treated cells, the numbers of completed chromosomes equal the numbers of origins present in a cell at the time of drug treatment (Skarstad et al. 1986, 1988). Flow cytometry analysis of the exponential samples reveals the DNA and protein content per cell at time of sampling and can be used to calculate DNA content-to-cell mass ratios.

As acidic phospholipid-depleted cells began to slow down in their growth, the number of origins per cell, as shown by rifampicin- and cephalexin-treated samples, decreased relative to cells that continued to synthesize acidic phospholipids (Fig. 1B vs. C, right columns). Specifically, at the first two time points, there was a distribution of four and eight origins in cells synthesizing acidic phospholipids (Fig. 1B; 1.5 and 2.75 h, right column), and by 5 h the distribution had shifted to eight and 16 origins, suggesting the cells had completely entered exponential growth (Fig. 1B; 5 h, right column). The distribution of origins in cells not synthesizing acidic phospholipids also was four and eight origins at the first two time points (Fig. 1C; 1.5 and 2.75 h, right column). However, as the cells approached arrested growth, the distribution shifted toward a majority of four origins (Fig. 1C; 5 h, right column).

Flow cytometry analysis of exponential samples revealed that cells continually synthesizing acidic phospholipids had a DNA content ranging from about 3.5 to 7 chromosomes (Fig. 1B, left column). However, in cells not synthesizing acidic phospholipids, the DNA content decreased by approximately 20% (Table 1). The accumulation of cells with four fully replicated chromosomes can be seen as a slight peak in the DNA distribution (Fig. 1C; left column, black arrow). Taken together, these results suggest a loss of an adequate level of acidic phospholipids causes an inhibition of initiation of replication. Flow cytometry analysis also showed decreased cell mass (Table 1). Therefore, even though both DNA content and cell mass decreased as cells became depleted of acidic phospholipids and arrested for growth, the DNA content-to-cell mass ratio remained constant (Fig. 1D).

**Table 1:** DNA content and cell mass after cessation of *pgsA* induction

Time (h)	DNA content (%)	Cell mass (%)
1.5	97.3 ± 6.1	104.7 ± 4.2
2.75	96.1 ± 2.0	92.7 ± 4.5
5	82.0 ± 4.4	78.9 ± 5.6

Results, presented as a percentage relative to the values for cells expressing *pgsA*, are the average and standard deviation of three experiments as outlined in Figure 1.

### Stringent response is not associated with the growth arrest of acidic phospholipid-deficient cells

The simultaneous decrease in cell mass and DNA content that occurred as cells became arrested for growth, as evident by the constant DNA content-to-cell mass ratio (Fig. 1 and Table 1), suggests that a global stress-response pathway may have been activated. A possible candidate was the well-characterized stringent response as it was previously shown to be activated by changes in fatty acid metabolism (Battesti and Bouveret 2006), and thus, changes in the composition of phospholipid headgroups might induce this pathway as well.

Under certain nutritional or other stress stimuli, the stringent response leads to the expression of survival genes and arrested growth (Cashel 1969; Magnusson et al. 2005; Potrykus and Cashel 2008; Potrykus et al. 2011). The response is typified by the accumulation of the intracellular alarmones guanosine 5′-(tri)diphosphate, 3′-diphosphate [(p)ppGpp] that are produced by RelA and SpoT. For example, amino acid starvation activates RelA-dependent synthesis of pppGpp (Potrykus and Cashel 2008).

**Figure 2:**
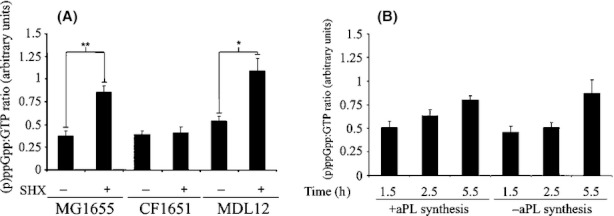
MG1655, CF1651, and MDL12 cells were grown at 30°C in MOPS minimal medium for indicated times. Radiolabeled guanosine nucleotides were extracted from cells, resolved by thin-layer chromatography on PEI cellulose plates, and visualized with a Storm 840 Phosphorimager. Analysis of three independent experiments. (A) Densitometric analysis of the relative combined amounts of pppGpp and ppGpp [(p)ppGpp)] versus GTP in cells before and after SHX treatment, and (B) in MDL12 cells expressing and not expressing *pgsA*. Error bars represent one standard deviation from the three independent experiments. *P-value <0.05, **P-value <0.005 (Student's *t*-test).

Wild-type MG1655 (*relA*^*+*^), CF1651 (*relA*^*−*^), and MDL12 cells grown in MOPS minimal medium that contained ^32^P-orthophosphate had doubling times of 54, 69, and 79 min, respectively (Fig. S1C and Table S1). The MG1655 and CF1651 strains served as controls for cells that can and cannot produce, respectively, *relA*-dependent (p)ppGpp (Metzger et al. 1989; Gaal and Gourse 1990). The cells were grown for two and a half hours and then treated with serine hydroxamate, an amino acid analog known to induce the stringent response by stalling protein translation and thus mimicking amino acid starvation (Tosa and Pizer 1971). Nucleotides were extracted from the cells, resolved by thin-layer chromatography, and visualized by autoradiography (see Experimental Procedures). Production of (p)ppGpp was clearly evident for the wild-type cells, but not detectible in the CF1651 cells, when treated with serine hydroxamate (Fig. 2A). The MDL12 cells, which are *relA*^+^, were capable of producing (p)ppGpp when challenged with serine hydroxamate (Fig. 2A). When acidic phospholipid synthesis was repressed in MDL12 cells (Fig. 2B), levels of (p)ppGpp were slightly increased after the cells arrested for growth at five and a half hours (Fig. 2B). However, the slight increase in (p)ppGpp production in MDL12 cells from 1.5 to 5.5 h was similar for cells synthesizing and not synthesizing acidic phospholipids (Fig. 2B). Thus, we conclude that repressed acidic phospholipid biosynthesis, and the resulting decrease in DNA content and cell mass as cells become arrested for growth, is not associated with induction of the stringent response.

### Restored growth to acidic phospholipid-deficient cells by expression of DnaA(L366K) is characterized by asynchronous initiation and uncoupled DNA content-to-cell mass ratio

**Figure 3:**
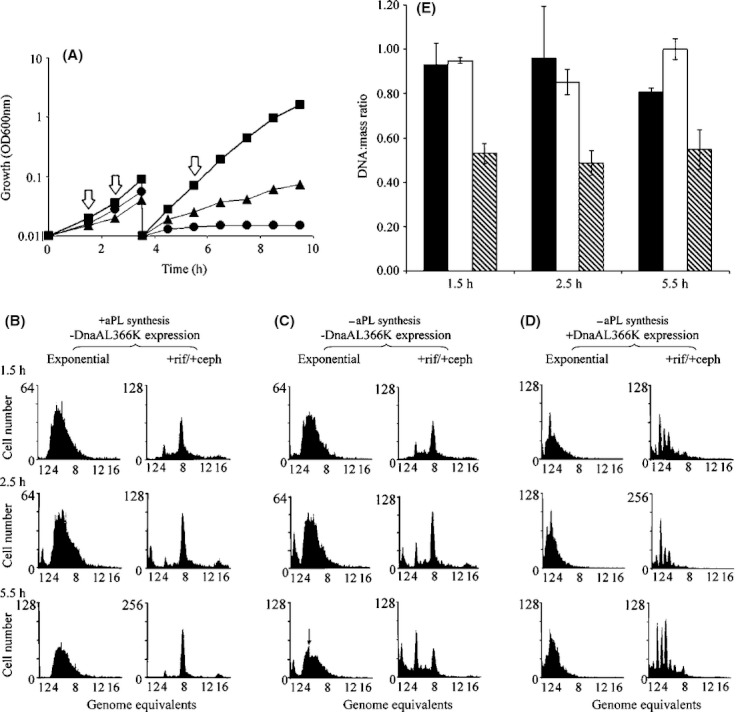
(A) MDL12/ZL607 cells were grown at 30°C in LB medium that contained kanamycin (50 μg/mL) and ampicillin (100 μg/mL), along with glucose (0.2%) (•, ▪), IPTG (1 mmol/L) (▪), or arabinose (0.2%) (▲). Open arrows indicate times that samples were collected for flow cytometry. At 3.5 h, the cultures were diluted with prewarmed fresh media to maintain exponential growth. (B–D) Flow cytometry histograms of exponential samples (left column) and rifampicin- and cephalexin-treated samples (right column) of cells (B) induced and (C and D) not induced for acidic phospholipid synthesis. (D) Cells were induced for the expression of DnaA(L366K). In panel C, the black arrow in the exponential histogram of the 5.5-h sample highlights the accumulation of cells with four fully replicated chromosomes. (E) DNA content-to-cell mass ratios, determined from FITC and Hoescht data from exponential samples, are an average of the ratio at a given time point, derived from three independent experiments for sample times 1.5, 2.5, and 5.5 h. Error bars represent one standard deviation. Black bars represent cells synthesizing acidic phospholipids, but repressed for DnaA(L366K) expression, white bars represent cells not synthesizing acidic phospholipids and repressed for DnaA(L366K) expression, and cross-hatched bars represent cells not synthesizing acidic phospholipids, but induced for expression of DnaA(L366K).

Expression of DnaA with a point mutation in the membrane-binding domain, DnaA(L366K), is known to restore growth to acidic phospholipid-deficient cells (Zheng et al. 2001). Thus, cells expressing DnaA(L366K) potentially represent a model of cell growth that is unregulated by acidic phospholipids. MDL12 cells were transformed with the plasmid pZL607 (Li et al. 2005), which has expression of DnaA(L366K) under control of the arabinose promoter (Guzman et al. 1995; Siegele and Hu 1997). MDL12/pZL607 cells cultured in LB medium in the presence of IPTG (1 mmol/L) and glucose (0.2%) to induce *pgsA* and repress DnaA(L366K) expression, respectively, continued to grow (Fig. 3A, filled squares), as seen earlier for nontransformed MDL12 cells induced with IPTG (Fig. 1A). The average doubling time under these conditions was 42 min (Fig. S1B, filled squares; Table S1). Flow cytometry analysis showed that the cells initiated their replication synchronously and possessed predominantly eight origins per cell at each time point examined (Fig. 3B, right column).

When the MDL12/pZL607 cells were not allowed to synthesize acidic phospholipids or DnaA(L366K), there was an initial period of growth before the culture became arrested for growth (Fig. 3A, filled circles). The onset of arrested growth occurred more rapidly than that observed in Figure 1; however, some variation was also observed in nonplasmid-bearing cells cultured from two independent colonies (Fig. S2). Flow cytometry analysis also showed the expected decrease from mostly eight to mostly four origins per cell (Fig. 3C, right column). Exponential samples of cells not induced for acidic phospholipid synthesis showed an accumulation of cells with four fully replicated chromosomes (Fig. 3C; 5.5 h, left column, black arrow). These results are consistent with those of nonplasmid-bearing cells (Fig. 1C; 5 h, left column, black arrow) and imply that initiation of chromosomal replication was inhibited.

However, if the loss of acidic phospholipids only inhibited initiation events, the distribution of peaks in the exponential sample of cells, which had ample time to normally complete ongoing rounds of replication after arresting for growth, would be similar to the profile of peaks for rifampicin- and cephalexin-treated cells; they would contain mostly completely replicated chromosomes. Yet, the flow cytometry profile of the exponential samples does not match the profile of the corresponding sample of cells treated with rifampicin and cephalexin. The exponential sample had a peak at four chromosomes, but still contained a proportion of cells with higher and lower DNA content (Fig. 3C, 5.5 h, left column). In the sample treated with rifampicin and cephalexin, we observed distinct peaks at four and eight origins (Fig. 3C; 5.5 h, right column), indicating that ongoing rounds of chromosomal replication, while prolonged in duration, could be fully resolved. Therefore, the decreased DNA content associated with deficient levels of acidic phospholipids may arise from a slowed average rate of DNA elongation as well as a decreased frequency of initiation events.

Wild-type *E. coli* cells initiate chromosomal replication simultaneously from all copies of *oriC* present once per cell cycle (Skarstad et al. 1986). Thus, synchronous initiations result in the generation of 2^*N*^ numbers of chromosomes per cell (*N* = 1, 2, 3…), with *N* depending on a cell's growth rate.

For cells not synthesizing acidic phospholipids, we observed that although they had fewer origins per cell as they approached growth arrest, their initiation events occurred synchronously (Fig. 3C; 5.5 h, right column). In contrast, cells that were already expressing DnaA(L366K), when shifted to conditions that repressed acidic phospholipid synthesis, continued to grow, albeit slower (Fig. 3A, filled triangles) with an increased doubling time of 104 min (Fig. S1B, filled triangles; Table S1). These cells initiated chromosomal replication asynchronously as indicated by the peaks of three, five, six, and seven genome equivalents (Fig. 3D, right column). Interestingly, acidic phospholipid-deficient cells expressing DnaA(L366K) consistently maintained a majority of two, three, or four origins despite the lack of continued acidic phospholipid synthesis. Of note, the minor shift in the number of origins in cells expressing DnaA(L366K) is likely due to entry of the cells into exponential growth rather than rifampicin insensitivity, as increasing concentrations of rifampicin had no effect on the number of origins per cells (Fig. S4). The fact that cells expressing DnaA(L366K) have similar flow cytometry profiles without regard to acidic phospholipid biosynthesis (*cf*. Fig. 3D, right column, 1.5 h vs. 5.5 h) implies that changes in acidic phospholipid content do not significantly affect the activity of DnaA(L366K). Indeed, similar asynchronous under-initiation of replication as seen here (Fig. 3D) was also seen for DnaA(L366K)-expressing *E. coli* cells possessing normal levels of acidic phospholipids (Zheng et al. 2001).

As was seen with the nontransformed cells, the DNA content-to-cell mass ratio was relatively constant in the presence (Fig. 3E, black bars) or absence (Fig. 3E, white bars) of acidic phospholipid synthesis when DnaA(L366K) expression was repressed. However, the DNA content-to-cell mass ratio was altered by the expression of DnaA(L366K) in acidic phospholipid-deficient cells, being substantially lower than that of both the growth-arrested and non-arrested MDL12 cells (Fig. 3E, cross-hatched bars). A similar decrease in DNA content-to-cell mass ratio was reported for wild-type cells expressing DnaA(L366K) (Zheng et al. 2001).

A decrease in this ratio could be caused by a decrease in the DNA content per cell, an increase in average cell mass, or a combination of both. For the acidic phospholipid-deficient cells expressing DnaA(L366K), the DNA content decreased relative to that in cells with normal acidic phospholipid levels, whereas their cell masses remained similar (Table 2). This is in agreement with the observation that there is under-initiation in cells expressing DnaA(L366K), including those whose growth is still preserved despite a loss in acidic phospholipids. Of note, the DNA content-to-cell mass ratios in cells expressing DnaA(L366K) sampled at 1.5, 2.5, and 5.5 h remained the same even though the cells sampled at 1.5 h would only have gone through one generation without *pgsA* expression.

**Table 2:** DNA content and cell mass after cessation of *pgsA* induction in cells either repressed or induced for the expression of DnaA(L366K)

	-*pgsA*, -DnaA(L366K)		-*pgsA*, +DnaA(L366K)	
		
Time (h)	DNA content (%)	Cell mass (%)	DNA content (%)	Cell mass (%)
1.5	100.2 ± 4.7	99 ± 16.1	67.3 ± 4.3	112.2 ± 13.6
2.5	84.8 ± 7.2	95.1 ± 20.9	54.5 ± 9.0	92.0 ± 31.6
5.5	81.7 ± 14.3	78.3 ± 27.5	63.0 ± 15.9	84.3 ± 16.7

Results, presented as a percentage relative to the values for cells expressing *pgsA*, are the average and standard deviation of at least three experiments as outlined in Figure 3.

### Restoration of acidic phospholipid synthesis results in a burst of DNA replication followed by cell division

The adverse effect on DNA replication and cell mass associated with the growth arrest as cellular acidic phospholipids levels decrease (Figs. 1 and 3) may be due to a block in a cell-cycle-specific event. To examine this possibility, we compared the number of cells and the rates of DNA synthesis of cells either: (i) continually synthesizing acidic phospholipids, (ii) not synthesizing acidic phospholipids, or (iii) initially not synthesizing acidic phospholipids until cell growth is arrested, followed by reinduction of *pgsA* expression.

**Figure 4:**
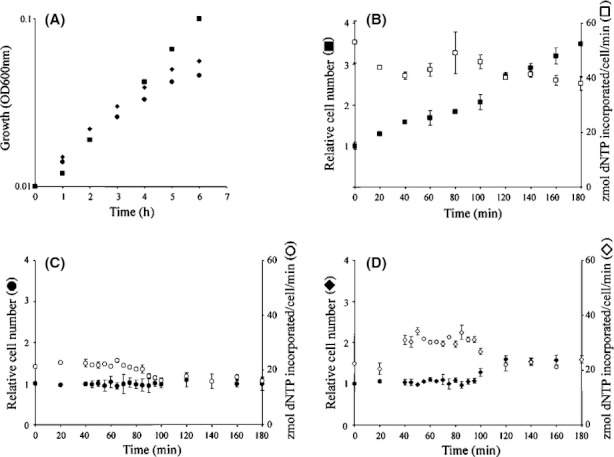
(A) MDL12 cells were cultured at 30°C in M9 minimal medium that contained glucose (0.1%), kanamycin (50 μg/mL), casamino acids (0.2%) and either with (▪) or without (•, ♦) IPTG (1 mmol/L), and growth was monitor by measurement of optical density (OD_600nm_). At 6 h, cells were harvested and suspended in fresh prewarmed media to an optical density of 0.025 (OD_600nm_) and used to assess DNA synthesis and cell growth (B–D). (B–D) The number of cells/mL at each time point is expressed as a ratio (▪, •, ♦) relative to the respective number of cells at 0 min (corresponding to the harvested and suspended cells after the sixth hour of culturing shown in panel A). The zmoles of dNTPs cell^−1^ mL^−1^ incorporated into acid-insoluble material in cells (B) continually synthesizing acidic phospholipids (□)*,* (C) not induced to synthesize acidic phospholipids (○), or (D) induced to synthesize acidic phospholipids after the initial 6 h of culturing (0 min of panel D) (◊). Samples were taken in triplicate for measurements of dNTP incorporation and number of cells, with average values displayed and error bars representing one standard deviation.

Three cultures, one of cells expressing *pgsA* and two that were not, were grown in M9 minimal media for 6 h, and growth was monitored by optical density (Fig. 4A). We had previously established that MDL12 cells, when grown in this medium in the absence of IPTG, exhibit a growth-arrest phenotype by 6 h (Fig. S3). The average doubling time of the *pgsA*-expressing cells was 78 min (Fig. S1D and Table S1), whereas the non-*pgsA*-expressing cells gradually ceased to grow. The substantial increase in doubling time in minimal media (M9) compared with LB medium may be related to increased cell lysis or media sensitivity, as observed in other viable *pgsA-*deficient strains (Kikuchi et al. 2000; Shiba et al. 2004). At the end of the 6-h period, aliquots of the cultures were used to inoculate prewarmed media to an optical density (OD_600nm_) of 0.025. Specifically, the culture that had grown in the presence of IPTG was used to inoculate medium containing IPTG (Fig. 4B), while one culture grown without IPTG was used to inoculate medium lacking IPTG (Fig. 4C), and the other uninduced culture was used to inoculate medium containing IPTG (Fig. 4D). For each of these cultures, samples were collected at various time points and numbers of cells were determined using a hemocytometer and a phase contrast microscope (Fig. 4B–D, filled symbols). To measure rates of DNA synthesis, samples of each culture were pulse labeled with radiolabeled thymidine for 3 min, mixed with ice-cold trichloroacetic acid, and incorporation of radiolabel into acid-insoluble material measured by liquid scintillation (Fig. 4B–D, open symbols).

During the final 3 h of culturing, the cells that had been continuously synthesizing acidic phospholipids showed a fairly constant rate of DNA synthesis of approximately 43 zmol dNTP incorporated per cell^−1^ min^−1^ (Fig. 4B). The cell density also increased at a consistent rate as expected for an asynchronous population of growing cells (Fig. 4B).

The culture in which *pgsA* remained unexpressed had a much different profile (Fig. 4C). There was no appreciable increase in cell number, consistent with arrested growth, and the rate of DNA synthesis was low, initially at approximately 22 zmol dNTP incorporated per cell^−1^ min^−1^, decreasing to 17 zmol dNTP incorporated per cell^−1^ min^−1^ after 100 min and onward (Fig. 4C). The lower rate of replication, relative to the *pgsA*-expressing culture, likely represented persistent chromosomal replication, with the decrease after 100 min suggesting completion of elongation activities. Indeed, cells expressing *pgsA*, but incubated with chloramphenicol (200 μg/mL; 180 min prior to radiolabeling) to block initiation of replication from *oriC*, incorporated a background signal of approximately 18 zmol dNTP per cell^−1^ min^−1^ (Fig. S5B). The presence of DNA synthesis in growth-arrested cultures prior to 100 min is marginal, but significantly above the chloramphenicol control, potentially reflecting the prolonged duration to resolve DNA replication due to lower levels of acidic phospholipids.

When *pgsA* expression was restored to growth-arrested cells, there was no change in the cell number or the rate of DNA synthesis for at least the first 20 min (Fig. 4D). However, by 40 min, a substantial increase in DNA synthesis was observed, which held constant at 31 zmol dNTP incorporated per cell^−1^ min^−1^ for approximately 1 h before returning to the initial value of approximately 22 zmol dNTP incorporated per cell^−1^ min^−1^ (Fig. 4D). Of note, the hour-long period of increased dNTP incorporation is commensurate with the time needed to typically complete ongoing rounds of chromosomal replication, and is thus suggestive that chromosomal replication occurs at a normal rate once acidic phospholipid synthesis is reinduced. The cell number remained nearly constant for 80 min, followed by an increase over the next 40 min, and then remained constant again for at least 20 min (Fig. 4D). The increases in cell number correspond with the commencement of significant increase in the optical density of the cultures, confirming that growth was occurring (Fig. S3A, filled diamonds).

**Figure 5:**
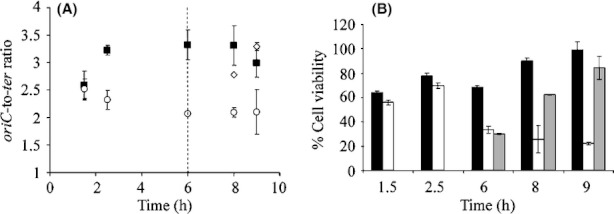
(A) MDL12 cells were cultured at 30°C in M9 minimal medium that contained glucose (0.1%), kanamycin (50 μg/mL), casamino acids (0.2%), and either with (▪) or without (○) IPTG (1 mmol/L) and maintained in exponential growth by dilution into fresh prewarmed media. After 6 h (dashed line), the culture without IPTG was split in half and IPTG (1 mmol/L) was added to one portion (◊) and incubation of the three cultures continued. Relative amounts of *oriC* and *ter* in extracted genomic DNA were determined by triplicate qPCR analyses. (B) Viability was assessed from cultures grown as described in panel A. The percentage of viable cells was assessed for (i) cells continually synthesizing acidic phospholipids (black bars), (ii) cells not synthesizing acidic phospholipids (white bars), and (iii) cells reinduced to express *pgsA* at 6 h (gray bars). The number of viable cells was determined by counting colonies from cultures plated onto LB agar plates containing IPTG (1 mmol/L) and kanamycin (50 μg/mL) and incubated for 24 h at 30°C. The total number of cells (viable and not viable) was determined by counting cells with phase contrast microscope and a hemocytometer. Viable and total counts were done in triplicate. The percent of viable cells represents the number of viable cells divided by the total number of cells. In both panels, error bars represent one standard deviation from the average.

Plating cells from the uninduced cultures at the 6-h time point (Fig. 4A) to test cell viability revealed that there is a proportion of cells in those cultures that are no longer viable (shown later in Fig. 5B), in agreement with the 60%, but not 100%, increase in the cell number at 120 min (Fig. 4D). The presence of a nonviable fraction of cells in the population may also explain why the dNTP incorporation rate in growth-restored cells did not increase to wild-type levels.

The burst in DNA synthesis followed by a step-wise increase in cell number suggest that the deficiency in acidic phospholipids caused a cell-cycle-specific block in the viable fraction of the cells, and that restoration of acidic phospholipid synthesis resulted first in resumed chromosomal replication followed by cell division.

### Loss of acidic phospholipids results in a reduction in *oriC*-to-*ter* ratio, and restoration of *pgsA* expression after growth-arrest restores the *oriC*-to-*ter* ratio

To confirm the thymidine labeling was indicative of DnaA- and *oriC-*dependent chromosomal replication and to further explore the possibility that decreased acidic phospholipid content affected the time required to complete chain elongation, an analysis of the abundance of DNA sequences (marker frequency) corresponding to *oriC* and *ter* was carried out using qPCR. Chromosomal DNA was recovered from cells grown in M9 minimal medium that were either: (i) continually synthesizing acidic phospholipids, (ii) not synthesizing acidic phospholipids, or (iii) initially not synthesizing acidic phospholipids until growth was arrested, followed by reinduction of *pgsA* expression.

Marker frequency was assessed with primers specific for *oriC* and *ter*. The *oriC* and *ter* signals were normalized to those from wild-type *E. coli* (MG1655) treated with rifampicin and cephalexin so as to have fully replicated chromosomes, and thus one copy each of *oriC* and *ter* per cell. In cells continually expressing *pgsA*, the *oriC*-to-*ter* ratio was 2.6 at 90 min after diluting the culture to an optical density (OD_600nm_) of 0.01, increased to 3.2 by 2.5 h, and remained between 3 and 3.3 for the duration of the experiment (Fig. 5A, filled squares). The initial increase in the *oriC*-to-*ter* ratio likely can be attributed to cells having fully entered exponential growth after the first couple of hours of growth.

The *oriC*-to-*ter* ratio in cells repressed for *pgsA* expression was approximately 2.5 after 1.5 h of growth (Fig. 5A, open circles), similar to that of the cells synthesizing acidic phospholipids. Under these conditions, both cells synthesizing acidic phospholipids and not synthesizing acidic phospholipids had a similar doubling time of 78 min (Fig. S3A and Table S1). By 2.5 h the ratio decreased to approximately 2.3 and to 2.0 by later times (Fig. 5A, open circles). Interestingly, while the ratio decreased relative to that of the cells continually synthesizing acidic phospholipids, it did not reach a ratio of 1:1, even after several hours of arrested growth. This result suggests that DNA elongation was inhibited or slowed as the cells became depleted of acidic phospholipids. Based on the ability of acidic phospholipid-deficient cells treated with rifampicin and cephalexin to complete chromosomal replication (Figs.1C and 3C) and the presence of a significant-but-lower level of DNA replication in acidic phospholipid-deficient cells (as measured by thymidine uptake; Fig. 4C), it is likely that the time for replicating the chromosome is increased rather than DNA elongation being directly inhibited. An assessment of cell viability revealed that 6 h after the initial dilution, cells lacking *pgsA* expression showed a 50–80% reduction in ability to form colonies on solid medium that contained IPTG, and the cell viability continued to decrease at later time points (Fig. 5B). Therefore, under these conditions, a portion of the chromosomal DNA analyzed by qPCR was obtained from nonviable cells, which likely contributed to the greater than 1:1 *oriC*-to-*ter* ratio in growth-arrested acidic phospholipid-deficient cells.

Six hours into the experiment, half of the culture not synthesizing acidic phospholipids was induced for acidic phospholipid synthesis by the addition of IPTG (Fig. 5A, vertical dashed line). By 2 h after addition of the inducer, the *oriC*-to-*ter* ratio in those cells had increased to approximately 2.8, and to 3.3 an hour after that (Fig. 5A, open diamonds). These increases restored the ratio in growth-rescued cells to that of the cells that had been continuously expressing *pgsA* (Fig. 5). Of note, by the time the ratio reached 2.75 (Fig. 5A; 8 h, open diamonds), it would have been well after the period of DNA synthesis following the reinduction of *pgsA* expression (Fig. 4D, 120 min). In summary, the qPCR data support the interpretation that the observed thymidine incorporation reflects restoration of DnaA- and *oriC*-dependent chromosomal replication, as replication initiated at random sites on the chromosome would have resulted in random duplication of *oriC* relative to *ter*.

## Discussion

The involvement of acidic phospholipids in the regulation of *oriC*-based chromosomal replication has been proposed for some time. In *E. coli*, *in vivo* studies have shown that acidic phospholipid deficiency leads to arrested growth (Heacock and Dowhan 1989), which can be restored by either bypassing *oriC*- and DnaA-dependent replication (Xia and Dowhan 1995) or by expressing certain mutant forms of DnaA protein (Zheng et al. 2001). Together, these findings suggest a link *in vivo* between acidic phospholipids and DnaA-dependent *oriC-*based chromosomal replication. In agreement with this model, we observed here that a deficiency in acidic phospholipids resulted in inhibited initiation of chromosomal replication (Figs. 1 and 3).

Acidic phospholipids, most notably cardiolipin, are known to reactivate replicatively inert ADP-DnaA to active ATP-DnaA *in vitro* by stimulating nucleotide exchange (Sekimizu and Kornberg 1988; Yung and Kornberg 1988; Crooke et al. 1992; Castuma et al. 1993). Inactive ADP-DnaA can only bind high-affinity DnaA “boxes” R1, R2, and R4 of *oriC*, while active ATP-DnaA can also bind low-affinity binding sites R3, R5, I1, I2 I3, τ1, and τ2 which is crucial in formation of prereplication complexes (Speck et al. 1999; McGarry et al. 2004; Kawakami et al. 2005; Leonard and Grimwade 2010). Thus, it has been speculated that one possible role *in vivo* for acidic phospholipids is to reactivate ADP-DnaA to replicatively active ATP-DnaA as a means to trigger prereplication complex formation.

It was previously seen for cells with normal levels of acidic phospholipids (Zheng et al. 2001) and here for cells with depleted levels (Fig. 3D) that expression of DnaA(L366K) results in under-initiation of chromosomal replication. This consistent under-initiation regardless of the cellular concentration of acidic phospholipids suggests that DnaA(L366K) activity *in vivo* is insensitive to changes in acidic phospholipids levels. However, it is worth noting that acidic phospholipids are depleted but not absent when cells not expressing *pgsA* become arrested for growth (Heacock and Dowhan 1989). Furthermore, purified DnaA(L366K) has been seen to require lower levels of acidic phospholipids than wild-type DnaA for *in vitro* nucleotide exchange (Aranovich et al. 2007). Thus, in determining the mechanism of how expression of DnaA(L366K) restores growth to the phospholipid-depleted cells, the possibility of membrane-mediated reactivation of ADP-DnaA to ATP-DnaA still needs to be considered a contributing mechanism. Exploration of the cellular levels of DnaA-ADP and DnaA-ATP in acidic phospholipid-depleted cells, in a similar manner as done by Kurokawa and colleagues (1999), would help assess this possibility. Other possibilities that need to be examined are whether DnaA or ATP concentrations are altered when acidic phospholipids are depleted. A drop in ATP concentration would be in agreement with a slowed rate of DNA elongation.

*In vitro* and *in vivo* studies have revealed that DnaA(L366K) is a feeble initiator of replication and cannot serve as the only form of DnaA in a cell (Zheng et al. 2001; Li et al. 2005). More recently, we observed that DnaA(L366K), whether in its ATP- or ADP-form, is unable to occupy the low-affinity sites of *oriC*, and likely participates as a heteromer with wild-type DnaA to initiate a round of replication (Saxena et al. 2011). The inefficient initiation activity of DnaA(L366K), even within a mixed oligomer with wild-type DnaA, may be the cause of asynchrony phenotype exhibited by DnaA(L366K)-expressing cells (Fig. 3D). The inefficiency of DnaA(L366K) to initiate replication might in part explain continued growth of cells depleted of acidic phospholipids. We have observed that cells expressing DnaA(L366K) consistently maintained a lower DNA content-to-cell mass ratio compared with cells with normal or depleted levels of acidic phospholipids (Fig. 3E). The inefficient activity of DnaA(L366K) may result in productive initiation events from only a subset of available origins, leading to asynchrony and a lower DNA content per cell.

Feeble initiation as a mechanism to suppress arrested growth in acidic phospholipid-deficient cells might entail preventing activation of a cellular shutdown pathway, similar to what has been observed with various DnaA mutants preventing activation of the SOS response caused by mutant DnaX_ts_ (Skovgaard and Løbner-Olesen 2005). However, we do not believe this is occurring here as several DnaA mutants are unable to restore growth to acidic phospholipid-deficient cells (Crooke and Zheng, unpubl. data). Still, it may be that acidic phospholipids are required for cell growth at a higher DNA content-to-cell mass ratio.

We observed a concomitant decrease in DNA content and cell mass when cells became deficient in acidic phospholipids, suggesting a global shutdown of cellular activity (Fig. 1 and Table 1). With respect to cellular lipids, the inhibition of fatty acid metabolism results in accumulation of (p)ppGpp (Seyfzadeh et al. 1993; Gong et al. 2002), and the (p)ppGpp-synthesizing protein SpoT is regulated by an important protein in the biosynthesis of fatty acids, acyl-carrier protein (ACP) (Battesti and Bouveret 2006). These results are consistent with a model that the stringent-response pathway may be involved in sensing differences in the fatty acid status of the cell (DiRusso and Nystrom 1998). We wondered whether the headgroup composition of the cell membrane phospholipids might also elicit the stringent response. Although the strain we worked with was capable of mounting the stringent response, we did not observe any appreciable generation of (p)ppGpp as cells became depleted for acidic phospholipids (Fig. 2), and therefore, it is unlikely that the stringent response is responsible for the shutdown in cellular activities that we observed. (p)ppGpp acts as a modulator of various sigma factors, influencing the transcription of many genes culminating in a survival response (Magnusson et al. 2005). While there is no evidence that DnaA has a broad role as a transcription factor triggering a survival response, DnaA has been shown to act as a transcription factor for its own promoter as well as other promoters (Atlung et al. 1985; Braun et al. 1985; Lother et al. 1985; Theisen et al. 1993; Ogawa and Okazaki 1994; Speck et al. 1999; Gon et al. 2006; Saxena et al. 2011). Thus, DnaA or other unknown factors may signal a similar biosynthetic shutdown in response to acidic phospholipid deficiency.

Another possible explanation of a global shutdown of cellular activity is that it is mediated by other stress-response pathways in a non-initiation-specific manner. Candidates include the Cpx two-component system and the σ^E^ pathway (Raivio 2005). Each has been documented to mediate a similar shutdown in cellular activity in response to certain stimuli. The Cpx system can be activated on depletion of the zwitterionic phospholipid phosphatidylethanolamine (Mileykovskaya and Dowhan 1997). The σ^E^ pathway can be activated by perturbations in outer membrane protein biogenesis (Mecsas et al. 1993; Raivio 2005), and outer membrane Braun's lipoprotein is known to require phosphatidylglycerol for its maturation (Sankaran and Wu 1994). A mutation or deletion in the gene encoding for lipoprotein (*lpp*) restores growth to acidic phospholipid-depleted cells (Matsumoto 2001; Suzuki et al. 2002). Thus, both of these pathways might mediate a response to altered membrane composition caused by the loss of acidic phospholipids.

Shortly after restoration of acidic phospholipid synthesis in growth-arrested cells, cell growth recovered (Figs. 4D and S3A) and an hour-long period of DNA replication occurred, followed a short interval later by cell division (Fig. 4D), indicating that arrested growth occurred at a specific stage of the cell-cycle prior to initiation. These results are in agreement with previous studies that showed phospholipid synthesis cycles with peak activity at initiation of chromosomal replication (Pierucci 1979) and inhibition of phospholipid synthesis blocks DNA replication at initiation (Pierucci and Rickert 1985). An alternative possibility is that cell division is inhibited by occlusion of the unreplicated nucleoid. This model could be tested using cells lacking functional nucleoid occlusion factor SlmA.

Our data also suggest that acidic phospholipids may be required for efficient chromosomal replication beyond the initiation event. Flow cytometry histograms of exponential samples of cells that had been growth arrested for over an hour revealed that a fraction of the cells had partially replicated chromosomes (Fig. 3C). Yet, these cells were capable of completing replication, as aliquots sampled in parallel and treated with rifampicin and cephalexin for several hours had fully replicated chromosomes (Fig. 3C). Under acidic phospholipid-deficient conditions, cells also were capable of low, but significant, levels of thymidine incorporation (Fig. 4C). Finally, qPCR analysis of the *oriC* and *ter* content in acidic phospholipid-deficient cells revealed that the *oriC*-to-*ter* ratio was not 1:1 upon the onset of growth arrest (Fig. 5). A model consistent with these results is that acidic phospholipids may be needed *in vivo* for efficient DNA elongation in addition to initiation of replication. While there is no evidence to date of a requirement for acidic phospholipids in DNA elongation, there is evidence that acidic phospholipids are important for assembly and function of several protein complexes. In mitochondria, cardiolipin is crucial in the organization and function of oxidative phosphorylation complexes (Lange et al. 2001; Zhang et al. 2002; Pfeiffer et al. 2003; McKenzie et al. 2006; Sedlak et al. 2006; Shinzawa-Itoh et al. 2007). Purification of supercomplexes, dubbed “respirasomes,” is reduced when using mild detergents in cardiolipin-null mutant cells (Zhang et al. 2002; Pfeiffer et al. 2003), and incorporation of complexes III and IV into supercomplexes in yeast cells is dependent on adequate concentrations of cardiolipin (Zhang et al. 2005). In prokaryotes, cardiolipin has been suggested to serve as an organizational center for many cell-cycle and cell-division proteins (Mileykovskaya and Dowhan 2009). Therefore, acidic phospholipids may be important in the optimal organization or activity of proteins contributing to DNA elongation.

## Supporting Information

**Figure S1.** (A) MDL12 cells were grown in LB medium that contained kanamycin (50 μg/mL) and IPTG (1 mmol/L) (▪). At 2.75 h, the cultures were diluted with prewarmed fresh medium to maintain exponential growth. (B) MDL12/pZL607 cells were grown in LB medium that contained kanamycin (50 μg/mL) and ampicillin (100 μg/mL), along with arabinose (0.2%) (▲), or glucose (0.2%) and IPTG (1 mmol/L) (▪). At 3.5 h, the cultures were diluted with prewarmed fresh media to maintain exponential growth. (C) MG1655 (+), CF1651 (x), and MDL12 (▪) cells were grown in MOPS minimal medium supplemented as described in Experimental Procedures. (D) MDL12 cells were cultured in M9 minimal medium that contained glucose (0.1%), kanamycin (50 μg/mL), casamino acids (0.2%), and IPTG (1 mmol/L) (▪). At 6 h, the cells were harvested and resuspended in prewarmed fresh media at an optical density of 0.025 (OD_600nm_). All growth occurred at 30°C.

**Figure S2.** MDL12 cells were grown at 30°C in LB medium that contained kanamycin (50 μg/mL) and without (•) or with IPTG (1 mmol/L) (▪). At 2.75 h, the cultures were diluted with prewarmed fresh media to maintain exponential growth. Panels (A) and (B) are growth profiles of cells from two different overnight inoculums, grown in parallel under identical conditions.

**Figure S3.** (A) MDL12 cells were cultured at 30°C in M9 minimal medium that contained glucose (0.1%), kanamycin (50 μg/mL), casamino acids (0.2%), and either without (•) or with (▪) IPTG (1 mmol/L), and growth was monitor by measurement of optical density (OD_600nm_). At 6 h, cultures were back diluted to an optical density of 0.025 with prewarmed fresh media, and the culture without IPTG was split in half and IPTG (1 mmol/L) was added to one portion (♦) and incubation of the three cultures continued. (B) The number of cells/mL at each time point is expressed as the cell number (▪, •, ♦) relative to the respective number of cells at 0 min (corresponding to the harvested and suspended cells after the sixth hour of culturing shown in panel A).

**Figure S4.** MDL12/pZL607 cells grown in LB medium and at indicated times samples were treated with rifampicin and cephalexin. At each time point, three aliquots of cells were treated with varying concentrations of rifampicin: 300 μg/mL (the concentration used in experiments for Figs. 1 and 3), 600 μg/mL, and 1200 μg/mL. The DNA content per cell of 10,000 cells from each sample was measured by flow cytometry.

**Figure S5.** MDL12 cells were grown for 6 h at 30°C in M9 medium that contained glucose (0.1%), kanamycin (50 μg/mL), casamino acids (0.2%), and IPTG (1 mmol/L). The culture was used to inoculate fresh prewarmed IPTG-containing M9 medium without (A) or with chloramphenicol (200 μg/mL) (B) to an optical density (600 nm) of 0.025, and growth was continued at 30°C for 180 min (A and B). For each double *y*-axis plot, the left *y*-axis shows the number of cells/mL (▪,▲) relative to the number of cells at 0 min (corresponding to the time of inoculating to an optical density [600 nm] of 0.025). The right *y*-axes display the rate of deoxynucleotide triphosphate (dNTP) incorporation into acidic insoluble material (□, Δ) for cells not treated (A) or treated (B) with chloramphenicol. Samples were taken in triplicate for measurements of dNTP incorporation and determination of relative cell number, with average values displayed and error bars representing one standard deviation.

**Table S1.** Doubling time (min) of *Escherichia coli* strains in various media.
