# Supplementary figures and images for: Depletion of acidic phospholipids influences chromosomal replication in Escherichia coli
Source: Microbiologyopen. 2012 Nov 16;1(4):450–66. doi: 10.1002/mbo3.46 (PMC3535390; doi:10.1002/mbo3.46)

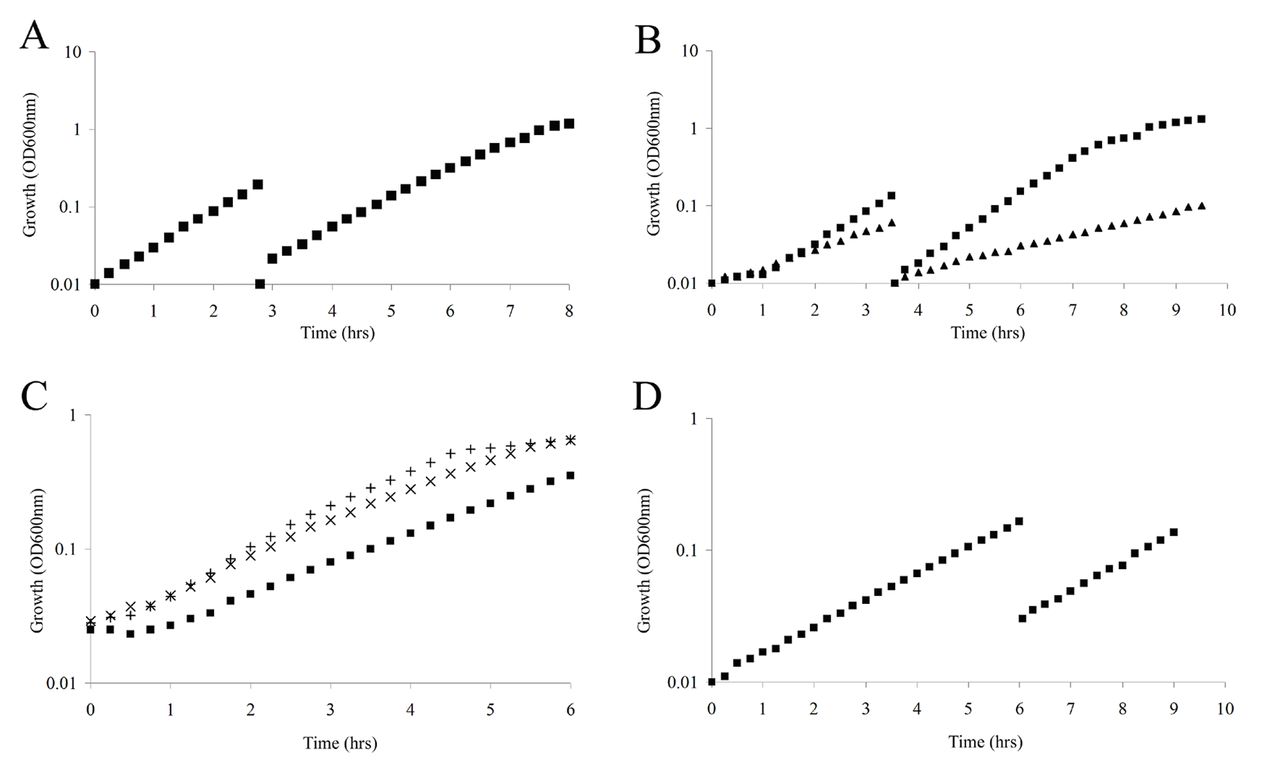

Supplement: Supplementary file 1 [file mbo30001-0450-SD1.jpg]

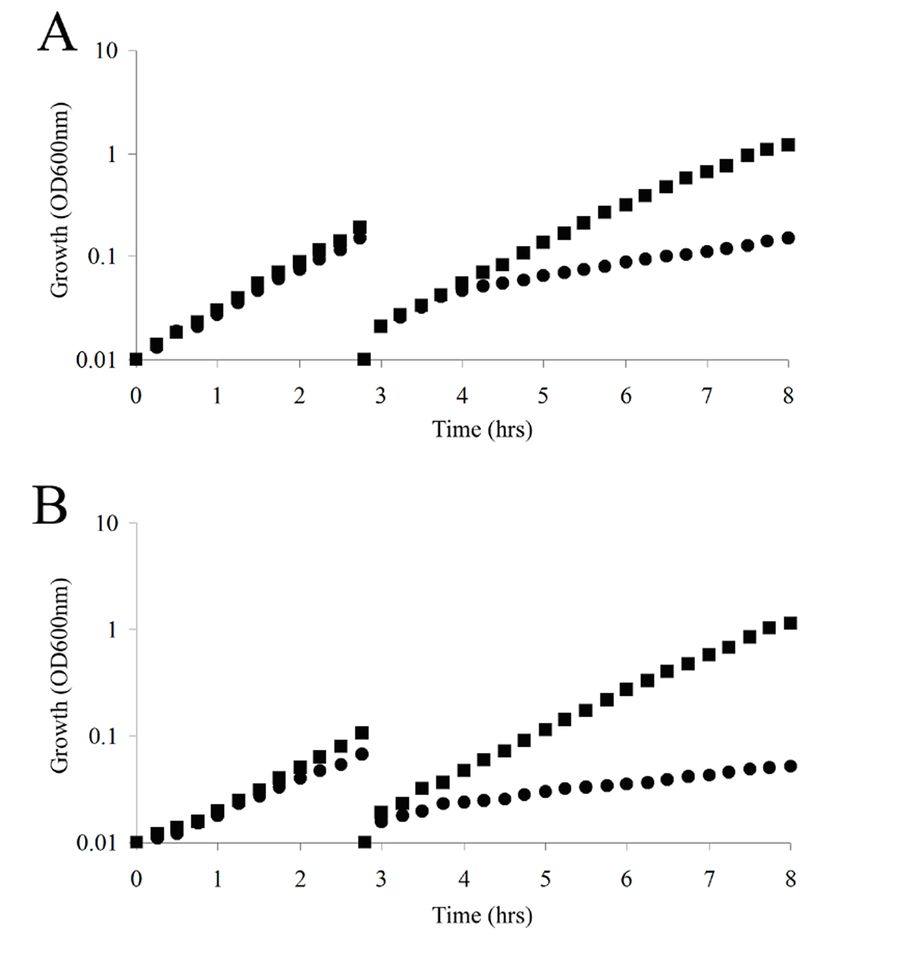

Supplement: Supplementary file 2 [file mbo30001-0450-SD2.jpg]

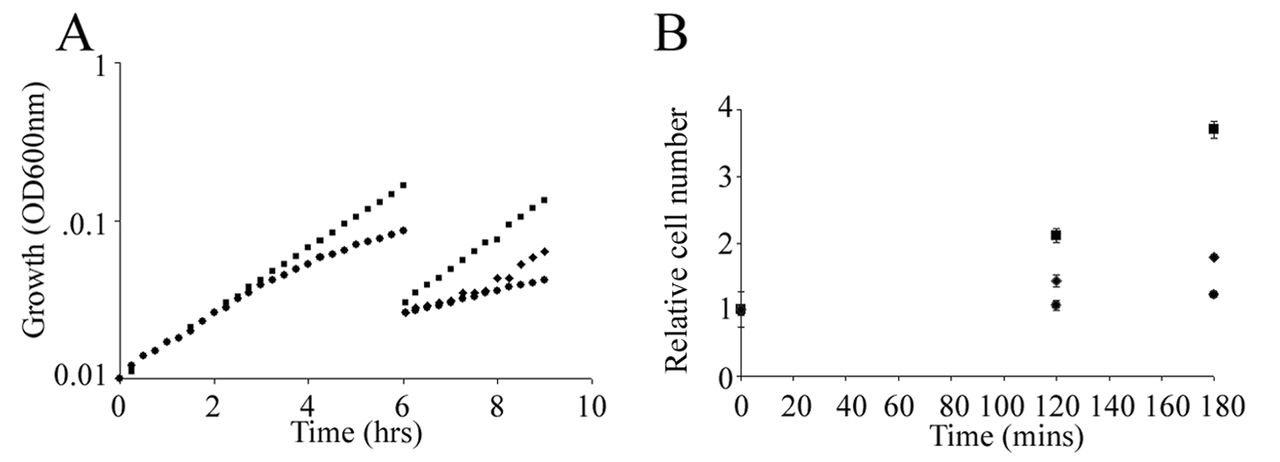

Supplement: Supplementary file 3 [file mbo30001-0450-SD3.jpg]

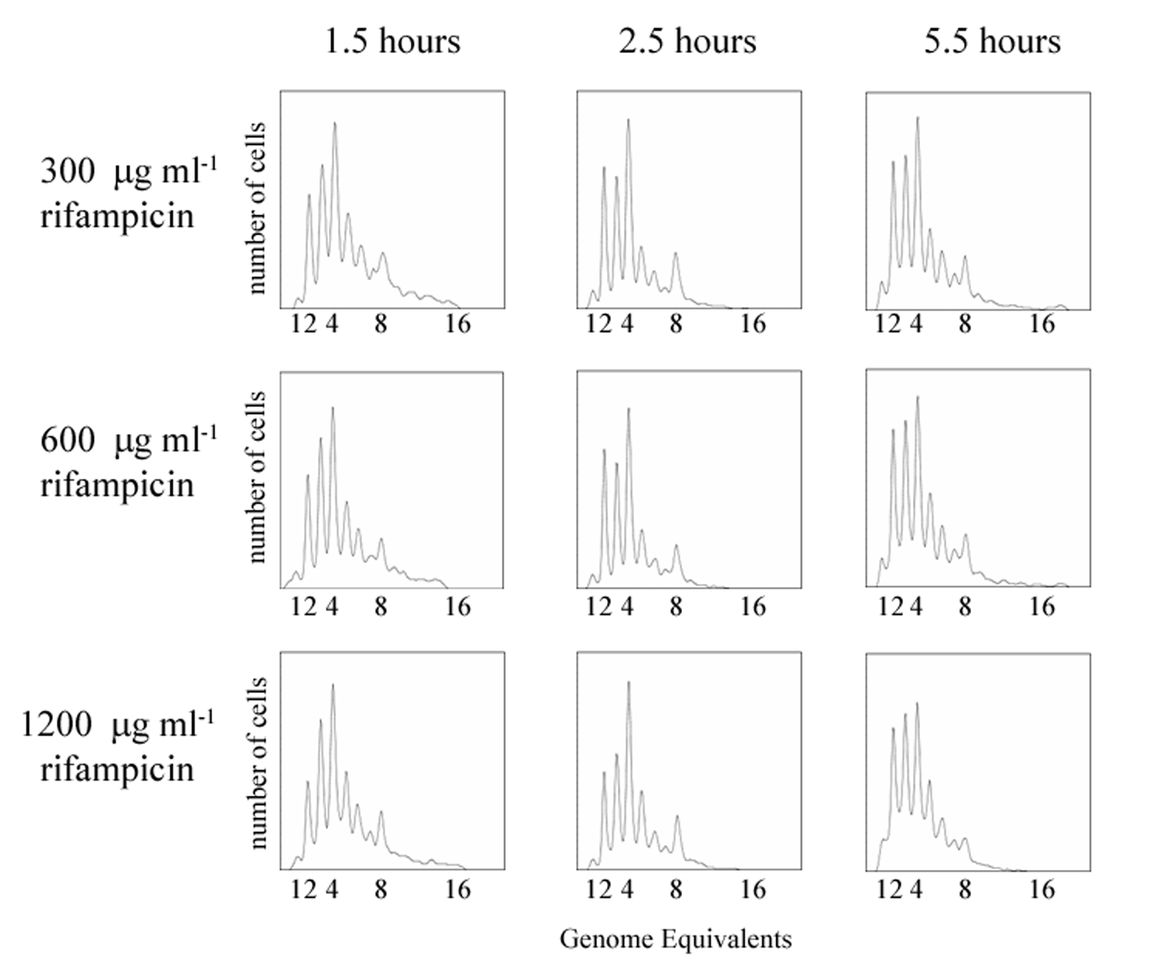

Supplement: Supplementary file 4 [file mbo30001-0450-SD4.jpg]

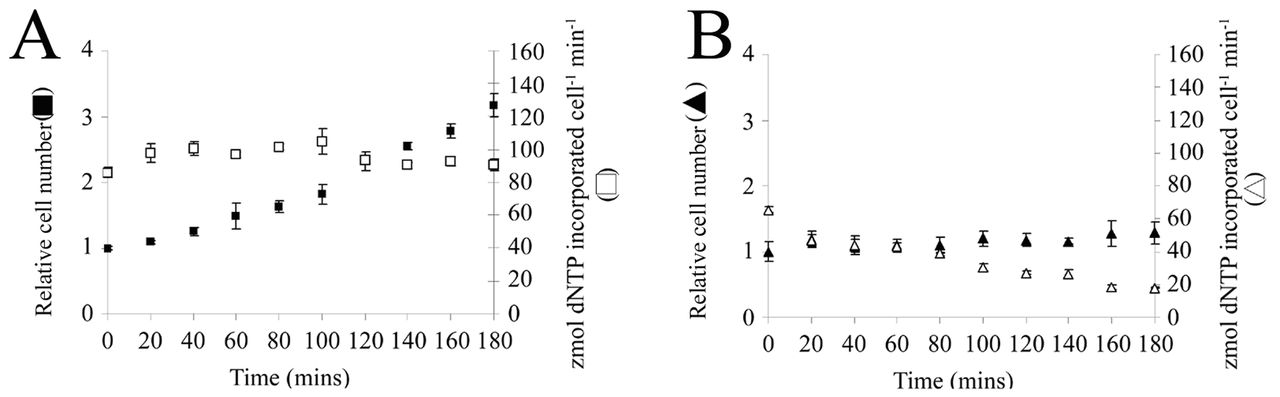

Supplement: Supplementary file 5 [file mbo30001-0450-SD5.jpg]
