# Supplementary material for: Depletion of acidic phospholipids influences chromosomal replication in Escherichia coli
Source: Microbiologyopen. 2012 Nov 16;1(4):450–66. doi: 10.1002/mbo3.46 (PMC3535390; doi:10.1002/mbo3.46)

Supplemental Table 1. Doubling time (min) of *Escherichia coli* strains in various media

| Strain | MOPS media | M9 media | LB media |
| --- | --- | --- | --- |
| MDL12 | 79 | 78 | 45 |
| MG1655 | 54 | ND^a^ | ND^a^ |
| CF1651 | 69 | ND^a^ | ND^a^ |
| MDL12/pZL607 | ND^a^ | ND^a^ | 42^+^, 104^*^ |

Values derived from optical density measurements (OD_600nm_) from experiments shown in supplemental figure 1. Doubling time was calculated as the length of time required to double in OD_600nm_ measurement during the period of steady-state exponential growth in each respective experiment. In each condition, cells were grown as described for the specific conditions in the experimental procedures. MDL12 growth curves were from cultures with 1mM IPTG to induce acidic phospholipid synthesis. ^+^ MDL12/pZL607 cells cultured with (0.2%) glucose to repress DnaA(L366K) expression. ^*^MDL12/pZL607 cells cultured with (0.2%) arabinose to induce DnaA(L366K) expression. ^a^ND, not determined.

Supplemental Figure Legends

Supplemental Figure 1. (A) MDL12 cells were grown in LB medium that contained kanamycin (50 µg ml^-1^) and IPTG (1 mM) (■). At 2.75 hours the cultures were diluted with pre-warmed fresh medium to maintain exponential growth. (B) MDL12/pZL607 cells were grown at in LB medium that contained kanamycin (50 µg ml^-1^) and ampicillin (100 µg ml^-1^), along with arabinose (0.2%) (▲), or glucose (0.2%) and IPTG (1 mM) (■). At 3.5 hours the cultures were diluted with pre-warmed fresh media to maintain exponential growth. (C) MG1655 (+), CF1651 (x) and MDL12 (■) cells were grown in MOPS minimal medium supplemented as described in Experimental Procedures. (D) MDL12 cells were cultured in M9 minimal medium that contained glucose (0.1%), kanamycin (50 µg ml^-1^), casamino acids (0.2%) and IPTG (1 mM) (■). At 6 hours cells were harvested and resuspended in pre-warmed fresh media at an optical density of 0.025 (OD_600nm_). All growth occurred at 30°C.

Supplemental Figure 2. MDL12 cells were grown at 30°C in LB medium that contained kanamycin (50 µg ml^-1^) and without (●) or with IPTG (1 mM) (■). At 2.75 hours the cultures were diluted with pre-warmed fresh media to maintain exponential growth. Panels (A) and (B) are growth profiles of cells from two different overnight inoculums, grown in parallel under identical conditions.

Supplemental Figure 3. (A) MDL12 cells were cultured at 30°C in M9 minimal medium that contained glucose (0.1%), kanamycin (50 µg ml^-1^), casamino acids (0.2%) and either without (●) or with (■) IPTG (1 mM), and growth was monitor by measurement of optical density (OD_600nm_). At 6 hours cultures were back diluted to an optical density of 0.025 with pre-warmed fresh media, and the culture without IPTG was split in half and IPTG (1 mM) was added to one portion (♦) and incubation of the three cultures continued. (B) The number of cells ml^-1^ at each time point is expressed as the cell number (■, ●, ♦) relative to the respective number of cells at zero minutes (corresponding to the harvested and suspended cells after the sixth hour of culturing shown in panel A).

Supplemental Figure 4. MDL12/pZL607 cells grown in LB medium and at indicated times samples were treated with rifampicin and cephalexin. At each time point, three aliquots of cells were treated with varying concentrations of rifampicin: 300 μg ml^-1^ (the concentration used in experiments for Figures 1 and 3), 600 μg ml^-1^, and 1200 μg ml^-1^. The DNA content per cell of 10,000 cells from each sample was measured by flow cytometry.

Supplemental Figure 5. MDL12 cells were grown for six hour at 30°C in M9 medium that contained glucose (0.1%), kanamycin (50 µg ml^-1^), casamino acids (0.2%) and IPTG (1 mM). The culture was used to inoculate fresh pre-warmed IPTG-containing M9 medium without (A) or with chloramphenicol (200 μg ml^-1^) (B) to an optical density (600nm) of 0.025, and growth was continued at 30°C for 180 minutes (A & B). For each double Y-axis plot, the left Y-axis shows the number of cells ml^-1^ (■,▲) relative to the number of cells at zero minutes (corresponding to the time of inoculating to an optical density (600nm) of 0.025). The right Y-axes display the rate of deoxynucleotide triphosphate (dNTP) incorporation into acidic insoluble material (□,∆) for cells not treated (A) or treated (B) with chloramphenicol. Samples were taken in triplicate for measurements of dNTP incorporation and determination of relative cell number, with average values displayed and error bars representing one standard deviation.

Supplemental Figure 1


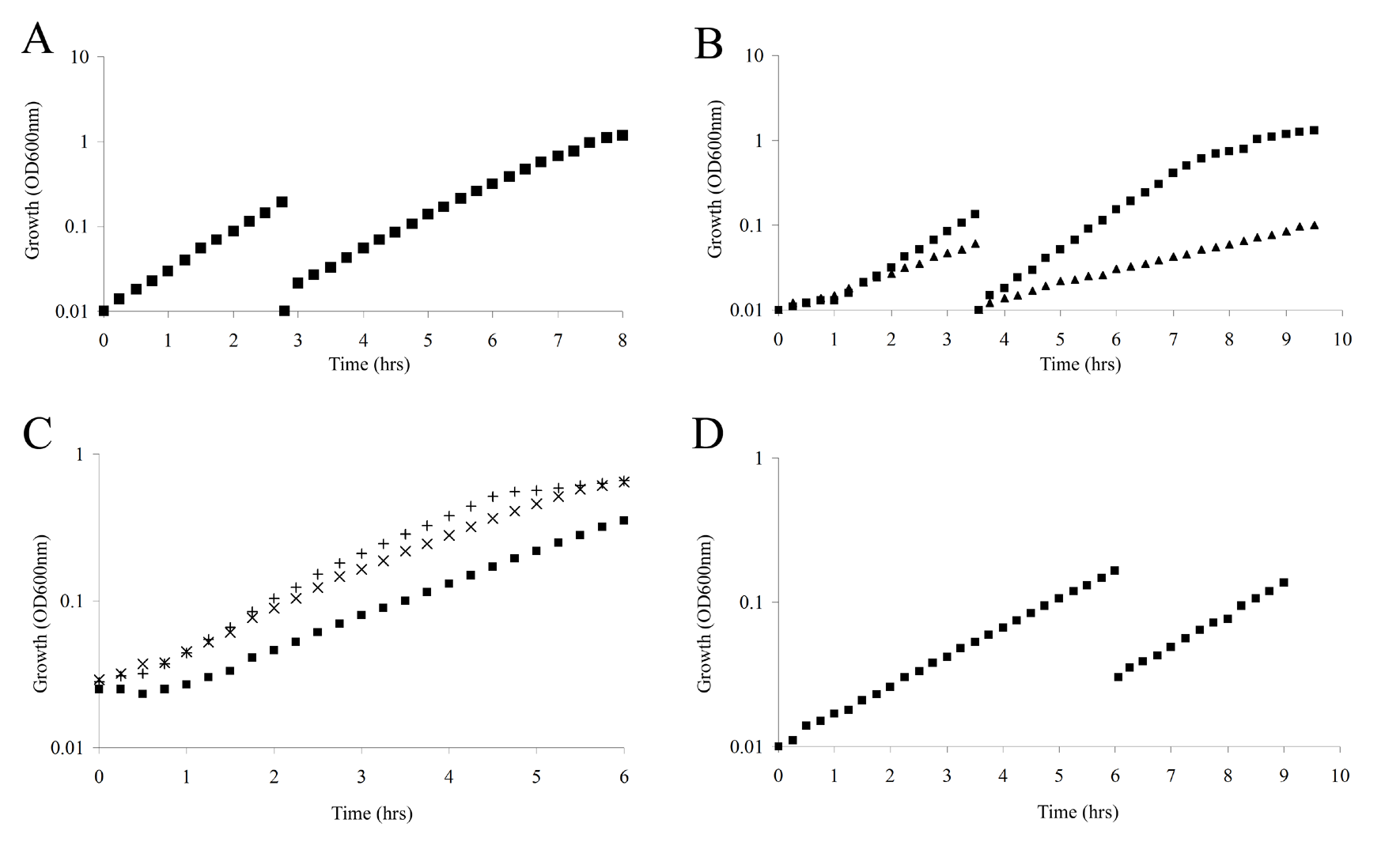


Supplemental Figure 2


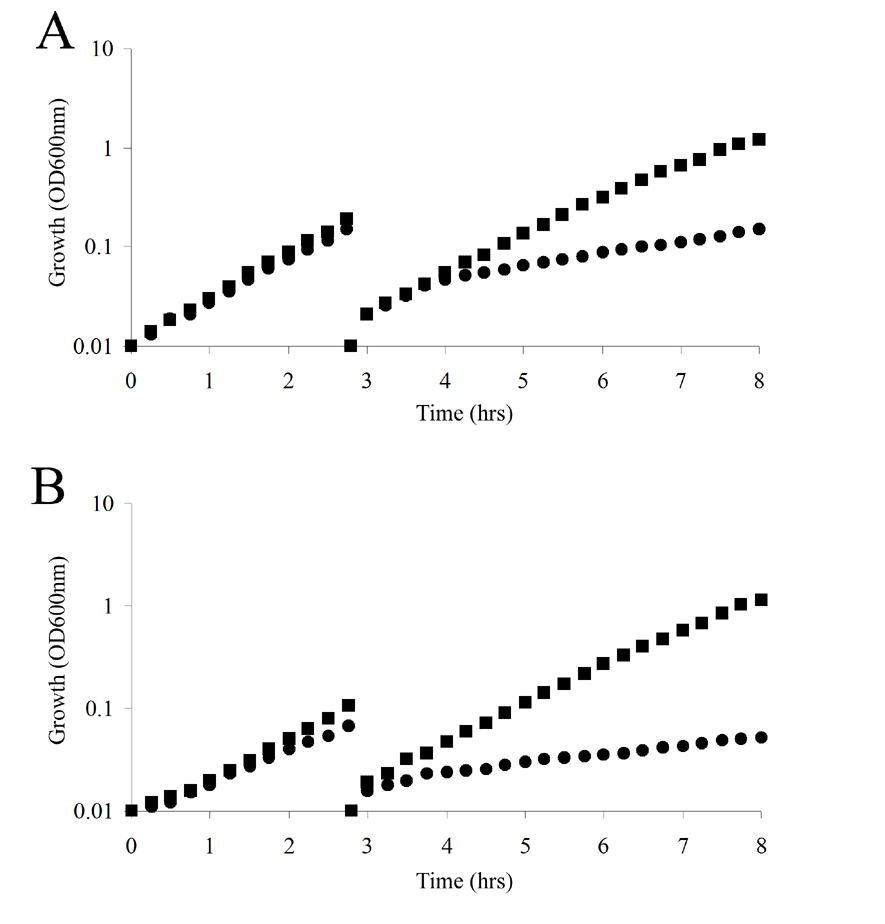


Supplemental Figure 3


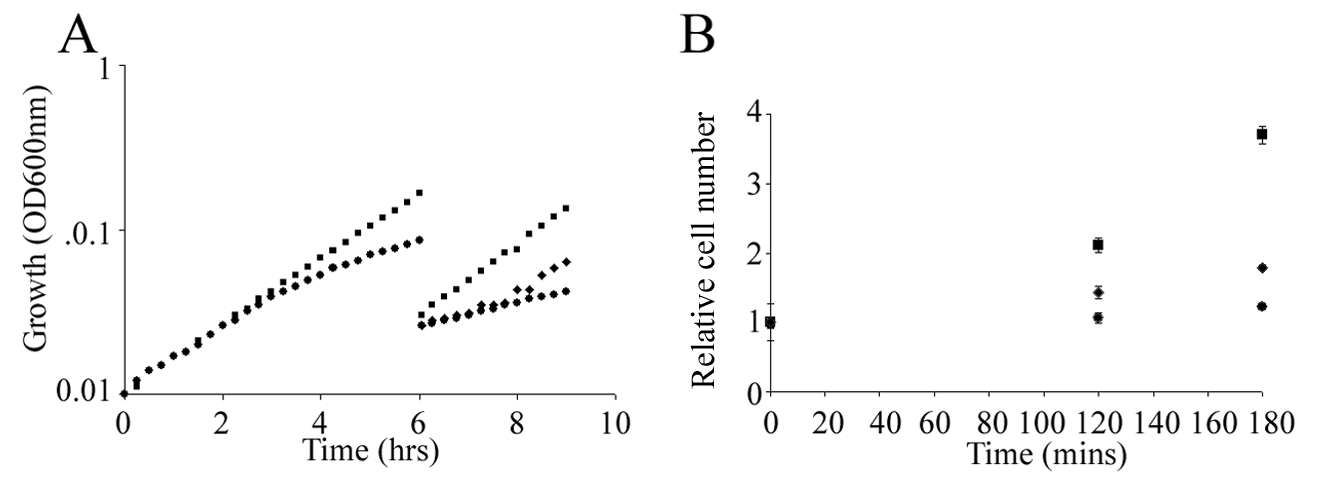


Supplemental Figure 4


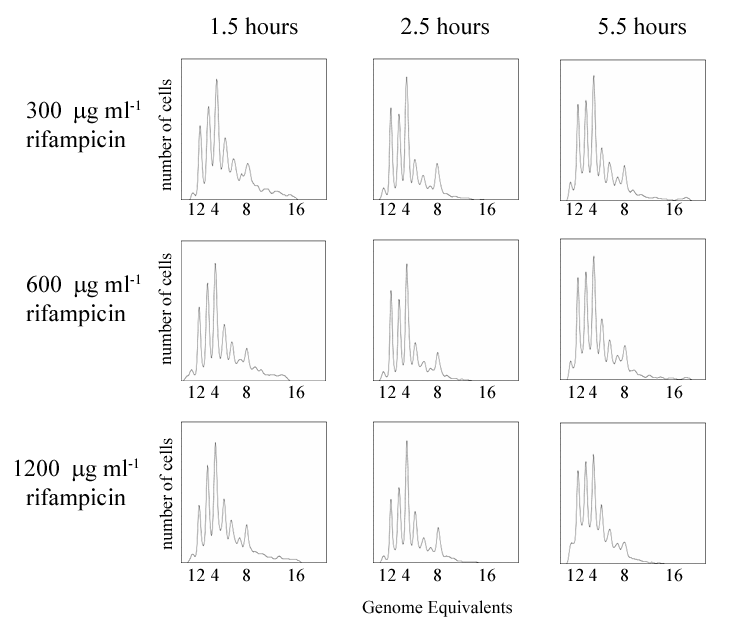


Supplemental Figure 5


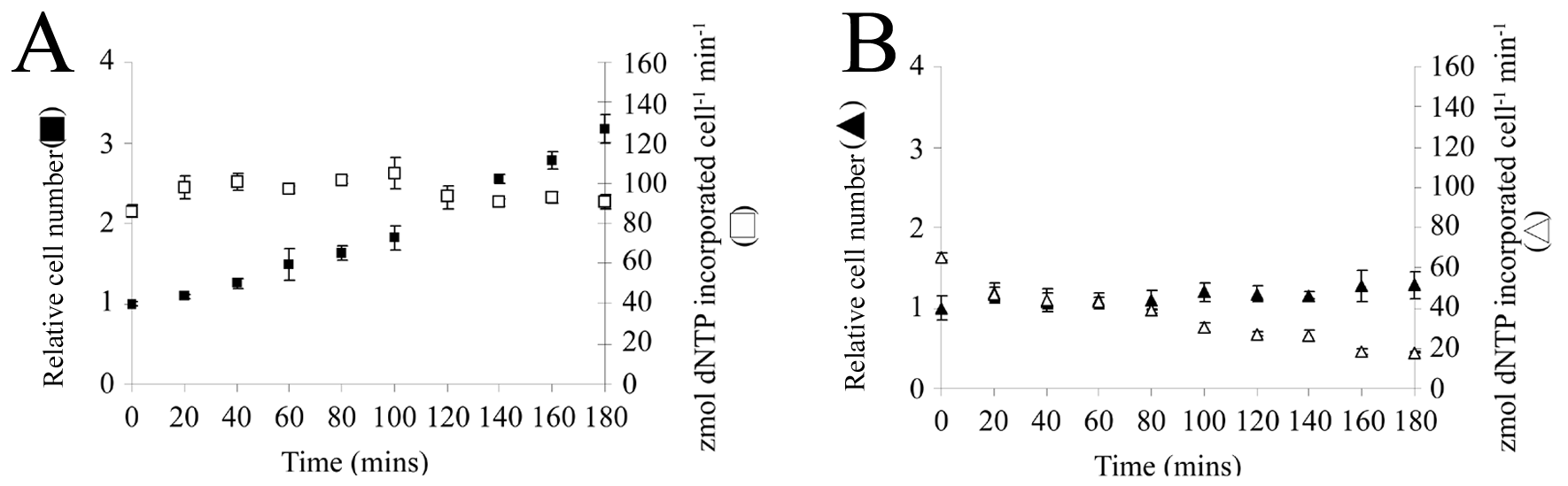

Supplement: Supplementary file 6 [file mbo30001-0450-SD6.docx]
